# Vaping Among Adolescents: An Overview of E-Cigarette Use in Middle and High School Students in India

**DOI:** 10.7759/cureus.38972

**Published:** 2023-05-13

**Authors:** Sairaj Khambayat, Arpita Jaiswal, Roshan Prasad, Mayur B Wanjari, Ranjana Sharma, Seema Yelne

**Affiliations:** 1 Medicine, Jawaharlal Nehru Medical College, Datta Meghe Institute of Higher Education and Research, Wardha, IND; 2 Obstetrics and Gynaecology, Jawaharlal Nehru Medical College, Datta Meghe Institute of Higher Education and Research, Wardha, IND; 3 Internal Medicine, Jawaharlal Nehru Medical College, Datta Meghe Institute of Higher Education and Research, Wardha, IND; 4 Research and Development, Jawaharlal Nehru Medical College, Datta Meghe Institute of Higher Education and Research, Wardha, IND; 5 Medical Surgical Nursing, Smt. Radhikabai Meghe Memorial College of Nursing, Datta Meghe Institute of Higher Education and Research, Wardha, IND; 6 Nursing, Shalinitai Meghe College of Nursing, Datta Meghe Institute of Higher Education and Research, Wardha, IND

**Keywords:** e-cigarettes, policy, health risks, prevention, vaping, adolescents

## Abstract

E-cigarette use among middle and high school students has become a growing public health concern in recent years. The prevalence of e-cigarette use among adolescents has increased dramatically, and there are serious health risks associated with this behavior. This review article provides an overview of e-cigarette use in middle and high school students, including the prevalence of use, contributing factors, health effects, policies and regulations surrounding e-cigarette use in schools, and interventions for preventing e-cigarette use in adolescents. The article highlights the need for effective prevention and cessation programs, increased public awareness about the risks of e-cigarette use, and stronger regulations on e-cigarette products. Addressing e-cigarette use among youth is critical for protecting the health and well-being of future generations, and it is important for parents, educators, healthcare providers, and policymakers to work together to prevent and reduce e-cigarette use among adolescents and promote healthy behaviors.

## Introduction and background

The use of e-cigarettes has been on the rise globally, and this trend has been particularly pronounced among adolescents [1]. The use of these devices is a significant public health concern because of the potential health risks associated with vaping, especially for young people. It is important to consider the local context to fully understand the impact and prevalence of this behavior, and in India, there has been a growing concern about the use of e-cigarettes among adolescents. The Indian government has responded to this concern by taking regulatory actions to curb the use of these devices. Therefore, it is important to examine the prevalence of e-cigarette use in India and explore the potential health effects of this behavior [2,3].

E-cigarettes work by heating a liquid solution that contains nicotine, flavorings, and other chemicals, producing an aerosol that is inhaled into the lungs. This process of inhaling the aerosol produced by e-cigarettes is commonly referred to as "vaping" [1,2] Initially marketed as a safer alternative to traditional tobacco products, concerns have been raised about the potential health risks of e-cigarette use, particularly in young people. Studies have shown that e-cigarette use can lead to addiction, respiratory problems, and other health issues [3,4].

The Centers for Disease Control and Prevention (CDC) and other health organizations have identified the use of e-cigarettes among adolescents as a public health concern. The long-term health effects of e-cigarette use are not yet fully understood, and the potential risks associated with vaping are a growing concern for public health officials. Therefore, it is important to raise awareness about the risks of e-cigarette use, particularly among young people, and explore ways to prevent and reduce the use of these devices [4].

This review aims to explore the various factors associated with e-cigarette use among adolescents, including social, cultural, psychological, and environmental factors, as well as the potential health consequences of vaping. The goal is to provide insights into the current state of e-cigarette use among young people and to identify gaps in the literature that need to be addressed in future research. Additionally, the review aims to inform policy and intervention strategies aimed at reducing adolescent vaping prevalence and related harms.

The purpose of this review article is to provide a comprehensive overview of e-cigarette use among middle and high school students in India. This will include an examination of the prevalence of e-cigarette use in this population, the potential health effects of e-cigarette use, and the policies and regulations surrounding e-cigarette use in schools. Additionally, we will explore the various interventions developed for preventing and ceasing e-cigarette use among adolescents in India.

The information presented in this review article is intended to synthesize the current research on e-cigarette use among adolescents in India and highlight the areas where further research and policy development are needed to address this growing public health issue. By thoroughly examining the various aspects of e-cigarette use among middle and high school students in India, this review article will serve as a valuable resource for educators, healthcare providers, policymakers, and others working to address this important public health concern in the Indian context.

## Review

Methodology

**Figure 1:**
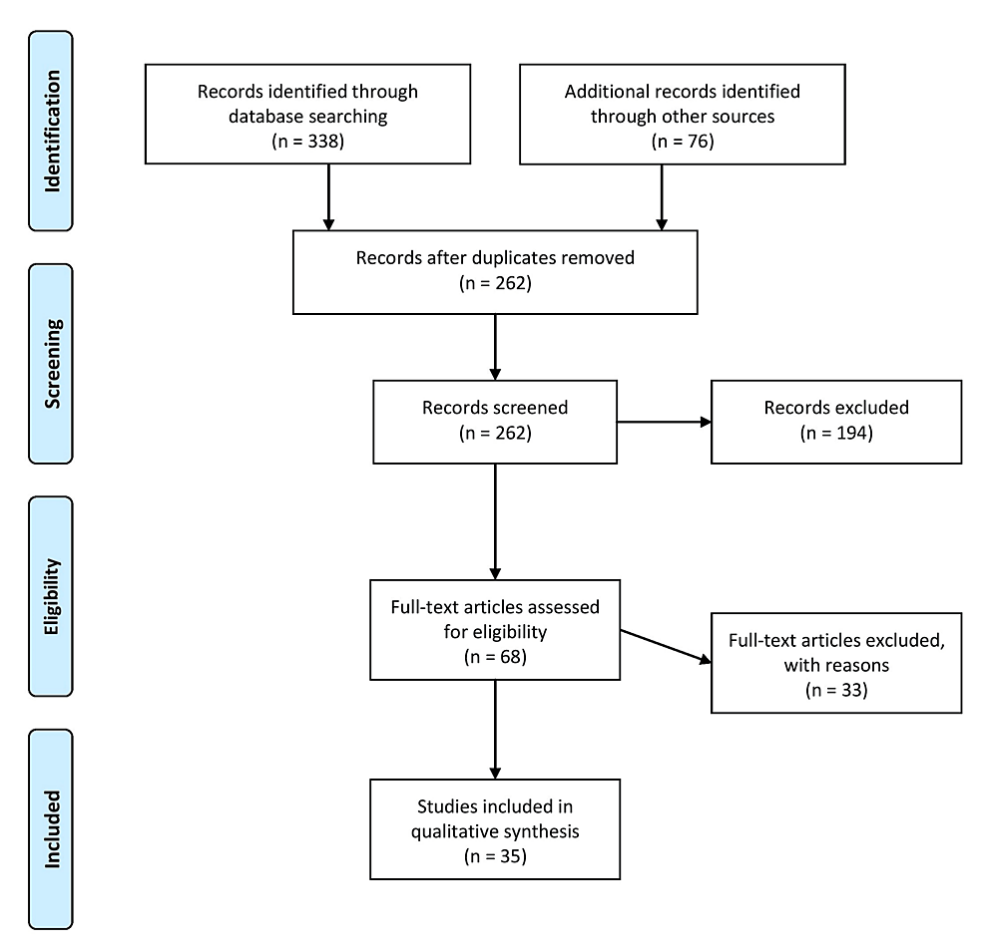
Selection process of articles used in this study. Adopted from the Preferred Reporting Items for Systematic Reviews and Meta-Analyses (PRISMA).

A comprehensive literature search was conducted using electronic databases such as PubMed, MEDLINE, PsycINFO, and Cochrane Library. The search encompassed articles published from 2010 to the present. It utilized specific keywords such as "e-cigarette," "vaping," "adolescents," "middle school," "high school," "prevalence," "risk factors," "health consequences," "nicotine addiction," and "smoking cessation." The articles were screened for relevance and eligibility based on inclusion and exclusion criteria. The inclusion criteria required that the articles be published in the English language, report on e-cigarette use among middle and high school students, report on the prevalence of e-cigarette use, associated factors, and potential health consequences, report on both observational and interventional studies, and published from 2000 to present, and that the articles were not duplicates. Figure 1 describes the selection process of articles used in our study.

Prevalence of e-cigarette use among middle and high school students

The use of e-cigarettes among middle and high school students has been on the rise in recent years. As per CDC, in 2019, the percentage of high school students using e-cigarettes increased from 1.5% in 2011 to 27.5% in the United States alone. Similarly, middle school students using e-cigarettes increased from 0.6% in 2011 to 10.5% in 2019 [4]. E-cigarette ever-use rose from 23% in 2015 to 37% in 2019 among Irish teenagers [5]. In 2015, the prevalence of “ever use” and “current use” of e-cigarettes in China was 3.1% and 0.5%, respectively. The lack of regulations for e-cigarette use and unrestricted practice encourage the increase in the adoption of e-cigarettes [6]. This global rise in e-cigarette use among adolescents is a worrisome trend that demands immediate attention.

Various factors contribute to the escalating trend of e-cigarette use among young people. One such factor is the widespread perception of e-cigarettes as a less hazardous alternative to traditional tobacco products. Advertisements for e-cigarettes commonly portray them as an effective means to quit smoking or as a healthier option than regular cigarettes. Furthermore, the abundance and ease of accessibility of e-cigarettes, especially those that come in a range of flavors, make them highly attractive to adolescents [7-10].

Marketing tactics employed by e-cigarette companies, such as social media marketing, sponsorships, and celebrity endorsements, have significantly contributed to the increase in e-cigarette use among adolescents. These strategies are often designed to target young people, normalize e-cigarette use, and increase the appeal of these products. For instance, social media platforms such as Instagram and Twitter promote images and messages that associate e-cigarette use with social status, attractiveness, and popularity [11,12]. Similarly, sponsorships and celebrity endorsements help create a positive image of e-cigarettes and make them seem more glamorous and desirable to young people.

As a result, the impact of e-cigarette marketing on adolescents cannot be overstated, as it influences their perceptions and attitudes toward these products. The use of social media influencers, who are often popular among adolescents, to endorse e-cigarette brands can be particularly influential in shaping young people's opinions. In addition, promoting flavored e-cigarettes that appeal to young people and using bright, colorful packaging also significantly attract adolescents to e-cigarettes [13,14].

Health effects of e-cigarette use in adolescents

The use of e-cigarettes among adolescents has been linked to various health risks. Among the most prevalent health concerns are respiratory and cardiovascular problems and the potential for nicotine addiction. E-cigarettes often contain harmful chemicals such as diacetyl and formaldehyde, which have been shown to contribute to lung disease and cancer. These risks underscore the importance of educating youth about the potential dangers of e-cigarette use and promoting healthy alternatives [15,16]. However, there are many potential health effects associated with e-cigarette use, particularly among this age group. Some of the health effects of e-cigarette use in adolescents are discussed below.

Nicotine Addiction

Nicotine addiction is a significant concern associated with e-cigarette use in adolescents. E-cigarettes typically contain nicotine, which is a highly addictive substance. Nicotine can quickly produce a pleasurable sensation in the brain, leading to a sense of reward and reinforcement. This can create a strong psychological dependence on nicotine, making it difficult for individuals to quit using e-cigarettes or other nicotine-containing products. Adolescents who use e-cigarettes and other nicotine-containing products are particularly vulnerable to developing addiction because their brains are still developing. Nicotine exposure during adolescence can alter brain development, leading to changes in the reward system and increasing the risk of developing addiction to other substances later in life [15].

Research has shown that nicotine exposure during adolescence can have long-term effects on brain function, including impairments in attention, learning, and memory. Furthermore, adolescents who use e-cigarettes are more likely to experiment with other substances, such as alcohol and marijuana, which can further increase the risk of addiction. Therefore, preventing and discouraging e-cigarette use among adolescents is critical to reducing the risk of nicotine addiction and associated negative health effects. This can be achieved through public education campaigns, regulatory policies, and comprehensive tobacco prevention and control programs [16].

Respiratory Problems

When an individual uses an e-cigarette, they inhale an aerosol that contains numerous chemicals and particles. Some of these chemicals and particles can be harmful to the respiratory system and can cause damage to the lungs. The particles in the aerosol are small enough to penetrate deep into the lungs, where they can cause irritation and inflammation. Over time, this can lead to respiratory problems such as coughing, wheezing, and asthma. In addition, some of the chemicals in e-cigarette aerosol have been linked to lung damage, including bronchiolitis obliterans, also known as "popcorn lung," a condition that causes scarring of the small airways in the lungs. Individuals who use e-cigarettes may be at an increased risk of developing respiratory problems, especially if they use these products regularly or over a long period. It is important for individuals to be aware of the potential risks associated with e-cigarette use and to seek medical attention if they experience any respiratory symptoms [17,18].

Cardiovascular Problems

The nicotine contained in e-cigarettes can negatively affect cardiovascular health, as it can increase the user's heart rate and blood pressure. This can strain the heart and blood vessels, increasing the risk of developing cardiovascular diseases such as heart disease and stroke. Long-term e-cigarette use with nicotine may also contribute to the development of atherosclerosis, which is the build-up of plaque in the arteries, further increasing the risk of cardiovascular disease. Additionally, the chemicals and particles in e-cigarette aerosol can cause inflammation and damage the blood vessels, contributing to cardiovascular problems. Therefore, adolescents must understand that using e-cigarettes with nicotine can harm their cardiovascular health and increase the risk of serious health problems later in life [19].

Mental Health Issues

There is growing evidence that e-cigarette use can negatively impact mental health, particularly among adolescents. Studies have found that adolescents who use e-cigarettes are more likely to report symptoms of anxiety and depression compared to their non-using peers. In addition, nicotine in e-cigarettes can affect neurotransmitters in the brain, impacting mood and increasing the risk of developing mental health issues. Furthermore, the act of vaping itself can also contribute to mental health issues. For example, repeatedly using e-cigarettes to cope with stress or anxiety can lead to an unhealthy dependence on the devices, exacerbating existing mental health issues or even developing new ones. It is also worth noting that there is a bidirectional relationship between mental health and substance use. Adolescents with pre-existing mental health conditions may be more likely to use e-cigarettes to cope with their symptoms, but, in turn, e-cigarette use can worsen their mental health [17-20].

Increased Risk of Substance Abuse

The use of e-cigarettes among adolescents has been associated with an increased risk of substance abuse. This means that adolescents who use e-cigarettes are more likely to use other tobacco products and illicit drugs, such as marijuana or cocaine. This is because nicotine, the addictive substance in e-cigarettes, can act as a gateway drug that leads to using other substances. Moreover, the social and environmental factors often accompanying e-cigarette use, such as exposure to peers who use other drugs and a tendency toward risk-taking behaviors, may also contribute to the increased risk of substance abuse [16-18].

Given the potential risks associated with e-cigarette use, parents, educators, and healthcare providers must educate adolescents about the potential health risks of using these products. This can include informing them about the addictive nature of nicotine and its potential impact on brain development, as well as the respiratory, cardiovascular, and mental health problems that can result from e-cigarette use. It is also important for parents and other trusted adults to discourage adolescents from using e-cigarettes and provide support and resources for those struggling with addiction or other related issues [19].

In addition to individual efforts, policies and regulations aimed at reducing youth access to e-cigarettes and restricting marketing and advertising of these products can also help prevent adolescent e-cigarette use and its associated health effects. This can include measures such as increasing the age for purchasing e-cigarettes, limiting the availability of flavored e-cigarettes that are attractive to youth, and implementing stricter regulations on marketing and advertising aimed at young people. Taking a comprehensive and multi-faceted approach can reduce adolescent e-cigarette use and its associated negative health effects [21].

Policies and regulations surrounding e-cigarette use in schools

In the United States, numerous federal and state laws regulate the use of e-cigarettes among young people. The Federal Food, Drug, and Cosmetic Act explicitly prohibits the sale of e-cigarettes to minors and mandates that manufacturers register their products with the FDA. Furthermore, several states have implemented legislation about e-cigarettes, which encompasses a variety of regulations such as sales restrictions to minors, limits on e-cigarette use in public areas, and taxes on e-cigarette products [22-24].

Schools have policies regarding e-cigarette use among students, with many incorporating e-cigarettes into their existing tobacco-free policies and prohibiting their use on school grounds. In addition to these policies, many schools have established enforcement measures to ensure compliance. For example, disciplinary actions may be taken against students caught using e-cigarettes on school premises, including suspension or enrolment in cessation programs. These measures are implemented to deter students from engaging in e-cigarette use, protect the health and safety of all students, and promote a healthy school environment. Schools must proactively prevent and reduce e-cigarette use among students and educate them about the potential health risks associated with these products [25-27].

Preventing e-cigarette use among adolescents requires the active involvement of parents and healthcare providers. Parents can play a crucial role by educating their children about the potential health risks associated with e-cigarette use and closely monitoring their behavior to detect any signs of vaping. Healthcare providers can also help by screening patients for e-cigarette use during routine medical visits, counseling patients and their families about the health risks of e-cigarette use, and providing evidence-based resources for smoking cessation. By working together, parents and healthcare providers can help prevent the uptake of e-cigarette use among adolescents and promote healthy lifestyles [28-30].

Interventions for preventing e-cigarette use in adolescents

Preventing adolescent e-cigarette use is an important public health goal, as e-cigarettes can harm developing brains and bodies. Several interventions are effective in preventing e-cigarette use in adolescents.

School-Based Programs

School-based programs incorporating e-cigarette education into the school curriculum are effective interventions for preventing adolescent e-cigarette use. These programs typically provide students with information on the risks and harms associated with e-cigarette use and strategies for avoiding e-cigarette use [31].

The educational component of these programs can include information on the various chemicals found in e-cigarettes, including nicotine, which is highly addictive and can harm adolescent brain development. The programs may also provide information on the potential long-term health consequences of e-cigarette use, such as lung damage, heart disease, and cancer [31,32]. In addition to providing education, school-based programs may teach students specific strategies for avoiding e-cigarette use. These strategies may include refusal skills, such as saying "no" to peer pressure to use e-cigarettes, and healthy coping mechanisms, such as stress reduction techniques, exercise, and mindfulness practices [32].

Parental Involvement

Parental involvement is a critical component in preventing e-cigarette use in adolescents. Parents can play a significant role in guiding their children toward healthier choices by actively participating in their lives. Studies have shown that parents who monitor their children's behavior and communicate regularly with them about the risks and harms associated with e-cigarettes are more likely to prevent their children from using these products [33].

One of the ways in which parents can monitor their children's behavior is by staying aware of their activities and social circles. By knowing who their children spend time with, parents can be more aware of potential negative influences that may encourage e-cigarette use. Parents can also have open and honest conversations with their children about the dangers of e-cigarettes and their potential long-term effects on their health. This includes discussing the addictive nature of nicotine and how it can lead to a lifetime of addiction [32].

In addition to monitoring their children's behavior and discussing the risks of e-cigarettes, parents can also set rules and boundaries around e-cigarette use. For example, parents can prohibit the use of e-cigarettes in their homes and establish consequences for violating this rule. They can also set clear expectations for their children's behavior and communicate the consequences of using e-cigarettes, such as losing privileges or facing disciplinary action [33].

Public Policies

Public policies aimed at restricting the sale and marketing of e-cigarettes to minors have been proven effective in preventing adolescent e-cigarette use. These policies can take several forms, such as age restrictions on e-cigarette sales, which make it illegal to sell e-cigarettes to anyone under a certain age. In the United States, the legal age to purchase e-cigarettes is 21, and many other countries have also established similar age restrictions [34,35].

In addition to age restrictions, policies can include restrictions on e-cigarette advertising. For example, policies may prohibit e-cigarette companies from advertising their products in places where minors are likely to be exposed, such as on television, billboards, or social media. These policies may also prohibit e-cigarette companies from using certain flavors, packaging, or labeling that could appeal to minors [31-33].

Another policy that has been implemented in some jurisdictions is taxing e-cigarettes. Taxes on e-cigarettes can make them more expensive and less accessible to young people with limited financial resources. This policy has been used successfully to reduce the use of traditional tobacco products in many countries and may also be effective in reducing e-cigarette use among young people [34].

Peer-Led Programs

Peer-led programs are initiatives that utilize the influence of peers to educate and influence other adolescents. These programs are effective in preventing e-cigarette use in adolescents. Peer-led programs can involve various activities such as peer-to-peer education and support, where trained peer educators provide information and guidance to their peers about the risks and harms associated with e-cigarette use. Peer-led interventions such as social media campaigns are another effective way to prevent e-cigarette use in adolescents [30-32].

In peer-led programs, young people are taught to recognize the potential dangers of e-cigarettes and communicate this knowledge to their peers in an engaging and relatable way. Peer educators are often trained to deliver messages using language and examples that resonate with their peers, which can be more effective than messages delivered by adults. Additionally, peer educators may be able to offer support and guidance to their peers in a way that is non-judgmental and supportive, which can help prevent e-cigarette use [33-35].

Peer-led programs can also take advantage of social media platforms to spread awareness and education about the harms of e-cigarette use. For example, peer-led social media campaigns may involve the use of popular platforms such as Instagram, Snapchat, and TikTok to disseminate information and raise awareness about the dangers of e-cigarettes. These campaigns can include user-generated content that is created and shared by young people themselves, which can be more effective in engaging and resonating with their peers [35].

Community-Wide Initiatives

Community-wide initiatives are programs or interventions that involve various stakeholders, such as schools, parents, public health officials, and community leaders, in the prevention of e-cigarette use in adolescents. These initiatives aim to create a collaborative effort among stakeholders to address e-cigarette use in adolescents and promote healthier behaviors [33].

One of the key benefits of community-wide initiatives is that they can involve a variety of interventions, including education, policy changes, and community outreach. For example, community-wide initiatives may involve educating parents and students about the risks of e-cigarette use, implementing policies that restrict the sale and marketing of e-cigarettes to minors, and conducting community outreach events to promote healthy behaviors [32].

It is important to note that community-wide initiatives may be most effective when used in combination with other interventions. For instance, a school-based program may be more effective when combined with parental involvement and public policies that restrict the sale and marketing of e-cigarettes to minors. By combining multiple interventions, community-wide initiatives can have a more comprehensive approach to preventing e-cigarette use in adolescents [31].

## Conclusions

In conclusion, e-cigarette use among middle and high school students is a significant public health concern that requires urgent attention. This review has highlighted the dramatic increase in the prevalence of e-cigarette use among adolescents and the serious health risks associated with this behavior. The findings underscore the urgent need for effective prevention and cessation programs, increased public awareness about the risks of e-cigarette use, and stronger regulations on e-cigarette products. Future research and policy should focus on developing and implementing evidence-based strategies to prevent and reduce e-cigarette use, promote healthy behaviors, and protect the health and well-being of our youth. Collaboration among parents, educators, healthcare providers, policymakers, and the community is essential in addressing this critical public health issue. By working together, we can ensure that future generations are protected from the harms of e-cigarette use and that our youth are able to thrive in a healthy and safe environment.
